# Strong Expression of Hypoxia-Inducible Factor-1α (HIF-1α) Is Associated with Axl Expression and Features of Aggressive Tumors in African Breast Cancer

**DOI:** 10.1371/journal.pone.0146823

**Published:** 2016-01-13

**Authors:** Hawa Nalwoga, Lavina Ahmed, Jarle B. Arnes, Henry Wabinga, Lars A. Akslen

**Affiliations:** 1 Centre for Cancer Biomarkers CCBIO, Department of Clinical Medicine, University of Bergen, Haukeland University Hospital, Bergen, Norway; 2 BerGenBio AS, Bergen, Norway; 3 Department of Pathology, Haukeland University Hospital, Bergen, Norway; 4 Department of Pathology, School of Biomedical Sciences, Makerere University College of Health Sciences, Kampala, Uganda; University of Tennessee Health Science Center, UNITED STATES

## Abstract

**Purpose:**

Inhibition of hypoxia-inducible factor (HIF) and Axl receptor tyrosine kinase is being evaluated for targeted therapy in solid tumors. Both HIF-1α and Axl influence tumor growth and metastatic potential, and they have been linked to treatment failure in many cancers. However, there is a lack of reports on HIF-1α expression in African breast cancer, which has a poor prognosis, and novel treatment targets must therefore be established. Here, we aimed to evaluate HIF-1α in relation to Axl expression, angiogenesis markers, and other tumor characteristics in a series of African breast cancer.

**Methods:**

Using immunohistochemistry, we examined 261 invasive breast cancers on tissue microarrays for HIF-1α and Axl as well as several other markers, and a subset of 185 cases had information on VEGF (vascular endothelial growth factor) expression, microvessel density (MVD), proliferating microvessel density (pMVD) and vascular proliferation index (VPI) for important comparisons.

**Results:**

Strong HIF-1α expression was associated with increased Axl (p = 0.007), VEGF (p<0.0005), and p53 (p = 0.032) expression, as well as high tumor cell proliferation by Ki-67 (p = 0.006), and high tumor grade (p = 0.003). Tumors with strong HIF-1α expression had significantly higher MVD (p = 0.019) and higher pMVD (p = 0.027) than tumors with weak expression.

**Conclusions:**

High HIF-1α expression is significantly associated with Axl and VEGF expression, and with markers of poor prognosis in this series of breast cancer, suggesting HIF-1α and Axl as potential therapeutic targets in African breast cancer.

## Introduction

Breast cancer is the most common malignancy affecting females worldwide, and it caused about 500,000 deaths in 2012, which is about 15% of all cancer deaths in women [1]. Metastases represent a major reason for cancer-related deaths; about 30% of breast cancer patients initially diagnosed with early-stage disease will eventually develop distant metastases [2]. Studies have shown that breast cancer is a heterogeneous disease, and understanding the molecular events that underlie this heterogeneity will lead to more precise and effective therapy. Regarding breast cancer in Africans and African Americans, previous studies have revealed that it has more aggressive features, is usually diagnosed in later stages, and has a poorer prognosis than breast cancer among Caucasians [3–5]. The reasons for this have not been fully characterized [4].

Tumor microenvironment factors have major influences on tumor development, growth and metastasis. As one factor, tumor hypoxia has been linked to aggressive phenotypes with associated chemoresistance and treatment failures in various cancer types, including breast cancer [6–9]. Hypoxia is also known as a key stimulus for angiogenesis, mainly via hypoxia-inducible factor 1 (HIF-1) [6, 9], which regulates transcription of several genes mediating tumor responses to hypoxia such as tumor cell proliferation, survival, migration and angiogenesis [6, 8]. During tumor hypoxia, HIF-1 is a main regulator of vascular endothelial growth factor (VEGF) and modulates angiogenesis by up-regulating the *VEGF* gene [6, 9, 10]. Vascular endothelial growth factor, one of the main factors responsible for the angiogenic switch during tumorigenesis, is a crucial mediator of angiogenesis in breast cancer [6, 8, 11]. Sustained angiogenesis is one of the hallmarks of cancer [12] and is a complex multi-step process, being essential for tumor growth, invasion and metastatic spread [6, 11, 13].

HIF-1α is a subunit of the HIF-1 heterodimer protein that is protected from degradation during the hypoxic response [6, 8, 14] when there is up-regulation of its mRNA with stabilization of the protein product and nuclear localization [6]. Previous evidence shows that HIF-1α is involved in breast tumorigenesis [15] and modifies tumor growth rates and their metastatic potential [6, 8, 9, 16]. Moreover, HIF-1α is over-expressed in about 24–56% of invasive breast cancers [17–21] or even more and has been associated with increased VEGF expression [15, 20], increased angiogenesis [21], higher tumor grade [15, 20], as well as treatment failure and poor prognosis [7, 19]. In experimental breast cancer models, resistance or sensitivity to EGFR-targeted therapies was dependent on HIF-1α activity in triple negative cell lines [22].

Several previous studies have revealed that hypoxia can independently stimulate the epithelial–mesenchymal transition (EMT) program, a critical step in cancer progression and metastasis, probably via a number of mechanisms [6, 8] such as HIF-1α signaling in several human tumors and cell lines: breast, pancreas, colon, kidney, lung and others [16, 23]. Furthermore, *in-vitro* and *in-vivo* studies have confirmed that hypoxia-induced EMT is tightly regulated by HIF-signaling pathways, which also contribute to additional tumor invasiveness by late release of VEGF, being mediated and sustained by HIF-1α [6, 8, 23]. Regulation of transcription factors Twist and ZEB1 has been shown to play a critical role in the hypoxia-mediated EMT process to promote metastasis [8, 16, 24].

Stimulation of EMT via transcription regulators such as Twist or ZEB1 has been associated with up-regulation of Axl, a tyrosine kinase receptor, in breast cancer epithelial cells [25], while a study of prostate cancer revealed that Axl expression was sustained in hypoxic tumor microenvironments [26]. Actually, Axl has been reported to have similar functions as HIF-1α in tumorigenesis such as promoting cell survival, migration, angiogenesis, invasion and metastasis among others [8, 25, 27]. According to previous studies, Axl might be a crucial regulator of EMT and is involved in the metastatic process in breast, prostate and lung cancers [26, 27], acting in both tumor cells and the supporting stroma [27, 28]. Previously, we reported a strong expression of Axl in breast cancer among African women [29].

Here, we aimed to explore HIF-1α expression in a series of African breast cancers in relation to Axl expression and other tumor characteristics. We found that strong HIF-1α expression was associated with increased Axl expression, markers of angiogenesis and other characteristics of aggressive tumors in this cohort of African breast cancer. Our results suggest that the therapeutic implications of HIF-1α and Axl co-expression in breast cancer should further be explored.

## Materials and Methods

### Patient series and specimens

This study was approved by the Research and Ethics Committee (REC) at Makerere University College of Health Sciences (MakCHS), Kampala, Uganda and the Regional Committee for Medical and Health Research Ethics (REC) of Western Norway (approval ID# 2014/1984/REK Vest). The REC at MakCHS waived the need for informed consent in accordance with the Uganda National Council for Science and Technology guidelines for conducting such a study. Initially, a total of 277 cases of primary invasive breast carcinoma with available archival paraffin blocks from the period 1990–2011 were identified as previously described from the records and archives at Department of Pathology, School of Biomedical Sciences at MakCHS, Kampala, Uganda [30]. Clinical information on the cases was obtained from histology reports, whereas information on treatment and follow-up was not available. Patient age ranged from 18 to 80 years (mean = 46 years, standard deviation 12.9). Tumor size was available in 60 patients and ranged from 1 to 20 cm (mean = 5.4 cm, standard deviation 3.2; median 5.0 cm). A majority of the tumors (50/60, 83%) were more than 2.0 cm in size. All cases were re-examined histologically and typed according to WHO recommendations [31], and histologic grading was performed in accordance with the Nottingham criteria [32]. Nuclear grade and mitotic count were recorded as separate variables using the same criteria [30].

### Immunohistochemistry

Tissue microarray (TMA) blocks were constructed, and 5 μm thick sections were made by standard technique and used for immunostaining as previously described [30]. We also included, for comparison, results for a subgroup of this series that had been previously stained (**Table 1**) for other biomarkers like estrogen receptor (ER), progesterone receptor (PR), human epidermal growth factor receptor 2 (HER2), Ki-67, p53, aldehyde dehydrogenase 1 (ALDH1), c-KIT, Cytokeratin 5/6, P-cadherin and epidermal growth factor receptor (EGFR) [33, 34] in addition to previous information regarding tumor-associated angiogenesis [35].

**Table 1:** Details of immunohistochemistry with antibodies and staining procedures.

Biomarker	Antibody	Clone	Dilution	Incubation time (min)
HIF-1α	MCM AH Anti-HIF-1 alpha antibody, (Santa Cruz Biotechnology Cat# sc-53546, RRID:AB_629639)	H1α 67	1:20	Overnight
Axl	Goat Anti-Human Axl Affinity Purified Polyclonal antibody (R and D Systems Cat# AF154, RRID:AB_354852)	Polyclonal	1:800	Overnight
Cytokeratin 5/6	MCM AH^a^ Cytokeratin 5/6 antibody (Dako Cat# M7237, RRID:AB_2281083)	D5/16 B4	1:200	30
EGFR	MCM AH Anti-EGFr Antibody, (Life Technologies Cat# 280005, RRID:AB_10835059)	31G7	1:30	30
P-cadherin	MCM AH P-Cadherin antibody (BD Biosciences Cat# 610227, RRID:AB_2077667)	56	1:400	60
ER	MCM AH Estrogen receptor α (Dako Cat# M7047, RRID:AB_2101946)	1D5	1:50	30
PR	MCM AH Progesterone receptor antibody (Dako Cat# M3569, RRID:AB_2532076	PgR 636	1:150	30
HER2	PCR AH c-erbB-2, c-neu antibody (Dako Cat# A0485, RRID:AB_2335701)	Polyclonal	1:500	60
Ki-67	MCM AH Ki-67 antibody (Dako Cat# M7240, RRID:AB_2142367)	MIB-1	1:50	60
p53	MCM AH p53 Tumor Suppressor Protein antibody (Dako Cat# M7001, RRID:AB_2206626)	DO-7	1:1000	60
VEGF	MCM AH VEGF antibody, (R and D Systems Cat# MAB293, RRID:AB_358222)	26503	1:20	60
**Previously stained markers** [30, 35]				
ALDH1	MCM AH Purified anti-Aldehyde dehydrogenase antibody (BD Biosciences Cat# 611195, RRID:AB_398729)	44	1:250	60
VPI	PCR AH Von Willebrand factor (Dako Cat# A0082, RRID:AB_2315602)& MCM AH Ki-67 antibody (Dako Cat# M7240, RRID:AB_2142367)	Polyclonal & MIB-1	1:800 & 1:50	60
c-KIT	PCR AH^b^ CD 117 (c-Kit, SCF-Receptor) antibody (Dako Cat# A4502, RRID:AB_2335702)	Polyclonal	1:200	30

VPI, vascular proliferation index.

^a^MCM AH, monoclonal mouse anti-human.

^b^PCR AH, polyclonal rabbit anti-human.

Regarding VEGF, HIF-1α and Axl staining, sections were deparaffinized in xylene, rehydrated through a series of graded alcohols and rinsed in distilled water. Antigen retrieval was achieved by microwave oven (MD 122, Whirlpool Nordic OY, Bromma, Sweden) heating in retrieval buffer (VEGF, Tris-EDTA pH 9.0; HIF-1α, citrate buffer pH 6.0) at 750 Watts for 10 minutes followed by 350 Watts for 15 minutes (an extra 20 minutes at 350 Watts was added for HIF-1α). For Axl, heating in target retrieval buffer pH6 (DakoCytomation [Dako], Glostrup, Denmark, S1699) in a 6^th^ Sense Jetchef Microwave Oven (JT 366, Whirlpool Nordic OY, Bromma, Sweden) for 25 minutes was utilized. All sections were allowed to cool at room temperature for 20 minutes, and then thoroughly rinsed in wash buffer solution. Additional staining was performed either by auto staining (VEGF and HIF-1α) in a Dako autostainer or manually (Axl antibody). Endogenous peroxidase activity was blocked by incubating sections with 0.03% hydrogen peroxidase (Dako, S2001) containing sodium azide for 5 minutes (VEGF and HIF-1α) or 15 minutes (Axl), followed by rinsing with wash buffer solution. In addition, to further reduce non-specific staining due to Axl antibody, we used the following: a background reducing antibody diluent (Dako, S3022) for dilution, a protein blocking serum (Dako, X0909) for 5–15 min before each step of antibody incubation, and rinsed the slides with several changes of wash buffer solution containing Tween 20 (Dako, S3306) in between the steps. Sections were incubated with specific antibodies either at room temperature (VEGF, dilution 1:20) or overnight at 4°C (HIF-1α, dilution 1:20; Axl, dilution 1:800). For Axl staining, a secondary rabbit anti-goat antibody from SouthernBiotech, Birmingham, AL (Catalog # 6164–01, dilution 1:400) was applied for 30 minutes at room temperature after three changes of wash buffer. Antigens were detected by incubating sections using appropriate Dako EnVision+ system-HRP kits (VEGF and Axl) or Dako Flex-EnVision kit (HIF-1α) for 30–35 minutes. After rinsing the sections in wash buffer solution, we developed the peroxidase by incubating with freshly prepared 3,3’-diaminobenzidine chromogen solution for 10 minutes (VEGF and HIF-1α) and 3 minutes (Axl). Sections were then rinsed in distilled water and counter stained with hematoxylin.

### Immunohistochemical assessment

A total of 16 tumors (5.8%) without interpretable cores containing sufficient tumor tissue were omitted from the analysis, and the remaining cases were evaluated for markers like HIF-1α, Axl, ER, PR, HER2, Ki-67 and p53 (**Table 1**). A subgroup of 192 tumors had information from previous studies [30, 33, 34] on VEGF expression, angiogenesis markers (see below), ALDH1 and c-KIT for important comparisons; 185 cases were fit for VEGF evaluation. For all markers, evaluation was done by visual microscopic assessment. Positive immunoreactivity was considered as follows (see also S1 Table): for HIF-1α and VEGF, nuclear and cytoplasmic staining were evaluated as being positive, respectively, while membrane and cytoplasmic staining was recorded for Axl; for HIF-1α cytoplasmic staining was observed in some cases but was not recorded; nuclear staining was assessed for ER, PR, Ki-67, p53 and c-KIT; cell membrane staining was considered for HER2 and EGFR; cell membrane and cytoplasmic expression was recorded for CK5/6 and P-cadherin; cell membrane and/or cytoplasmic staining was considered for c-KIT, as previously indicated [30, 33–35]; cytoplasmic staining was evaluated for ALDH1, while nuclear staining alone was considered nonspecific and was not recorded [30].

Staining was assessed by a semi-quantitative and subjective grading system that considers intensity of staining and proportion of tumor cells showing positive staining. For most of the markers, a staining index (values = 0–9) was determined by multiplying the score for intensity of staining (none = 0, weak = 1, moderate = 2 and strong = 3) with the score for proportion of tumor cells stained (<10% = 1, 10%–50% = 2, >50% = 3).

Cut-points for positive expression of the various markers are shown in **S1 Table**, as previously reported [33–35]. Briefly, the cut-off values for the SI categories used in statistical analysis were based on median or quartile values, also considering the frequency distribution and size of the subgroups. Based on the median staining index, the cut-off values were set at 0–2 = negative or weak expression and 3–9 = strong (high) expression for HIF-1α, VEGF, Axl and ALDH1 [30]. Similarly, a cut-point based on median SI for this series, SI = 0–3 as negative and SI = 4–9 as positive, was used for P-cadherin, whereas the upper quartile (SI = 0–4 as negative and SI = 6–9 as positive) was applied for p53 staining. Staining index for CK5/6 showed a high proportion of negative cases, and tumors were therefore categorized by SI = 0 as negative and SI = 1–9 as positive. For EGFR staining, tumors with any cell membrane staining, whether circumferentially complete or incomplete, observed in more than 1% of the tumor cells, were considered as positive [36]. Tumors were considered positive for ER, PR and c-KIT when at least 10% of the tumor cells were stained (weakly, moderately or strongly). Regarding HER2 status, cases were considered positive when the HER2 IHC score was 3+, whereas cases with 0–2+ scores were categorized as negative in this study. After determining the proportion (%) of Ki-67 positively stained nuclei out of 500 tumor cells counted at high power magnification (x400) using an eyepiece grid, the cut-point for high tumor cell proliferation rate by Ki-67 was set at 20.0% based on the median value as previously described [30].

Information on angiogenesis markers was available for a subset of the cases (n = 192) from previous studies and were included in the present study for important comparisons [35]. Briefly, tumor-associated angiogenesis was assessed by using dual staining with Factor VIII and Ki-67 antibodies, on regular tissue sections, as reported [35]. All positively stained vessels (red), within the hot-spots, most often in the tumor periphery, were counted including vessels without microlumina and clusters of endothelial cells clearly separate from adjacent microvessels in accordance to Weidner’s approach [37]. Dividing endothelial cells recognized by co-expression of Factor VIII and Ki-67: red cells (Factor VIII) with blue nuclei (Ki-67) were counted as proliferating microvessels [35]. Concisely, microvessel density (MVD) was taken as the number of vessels counted and expressed as MVD per mm^2^, while proliferating microvessel density (pMVD) was the number of microvessels with proliferating endothelial cells expressed per mm^2^. The percentage of pMVD (mm^2^) to the total MVD (mm^2^) was defined as vascular proliferation index (VPI). Altogether, 177 cases were fit for univariate statistical analysis using non-parametric tests; 15 cases (7.8%) were excluded due to poorly stained or insufficient tumor tissue [35].

### Molecular subtyping

Based on our previous studies [30], we defined various basal-like phenotypes (BLPs) as concurrent ER−, HER2− and: CK5+ as BLP1; P-cadherin+ as BLP2; EGFR+ as BLP3; CK5+ and/or EGFR+ as BLP4; and BLP5 as concurrent ER−, HER2− and positivity of at least one basal markers (CK5, P-cadherin and EGFR). BLP4 corresponds to the core basal phenotype described previously by Nielsen et al [38]. In addition, we determined molecular subtypes using immunohistochemistry in accordance with a slightly modified Goldhirsch et al criteria [39], where <20% = low and ≥ 20% = high were used as cut-points for Ki-67 score [29]. Thus, tumors were classified as follows: luminal A subtype (ER+ and/or PR+, HER2− and Ki-67 < 20%), luminal B subtype (luminal B, HER2 negative [ER+ and/or PR+, HER2− and Ki-67 ≥ 20%] and luminal B HER2 positive [ER+ and/or PR+ and HER2+]), HER2 subtype (ER−, PR− and HER2+), basal-like subtype (ER−, PR−, HER2− and CK 5/6+ and/or EGFR+) and unclassified category (ER−, PR−, HER2−, CK 5/6− and EGFR−).

### Statistical analysis

Statistical analysis was performed using the IBM SPSS Statistics for Windows, Version 22.0 (IBM Corp, Armonk, NY). Associations between different categorical variables were assessed using the Pearson’s χ^2^ test, while for quantitative data, the median values were compared using Mann-Whitney U test, and a p-value of < 0.05 was considered significant for any statistical test used.

## Results

**Fig 1:**
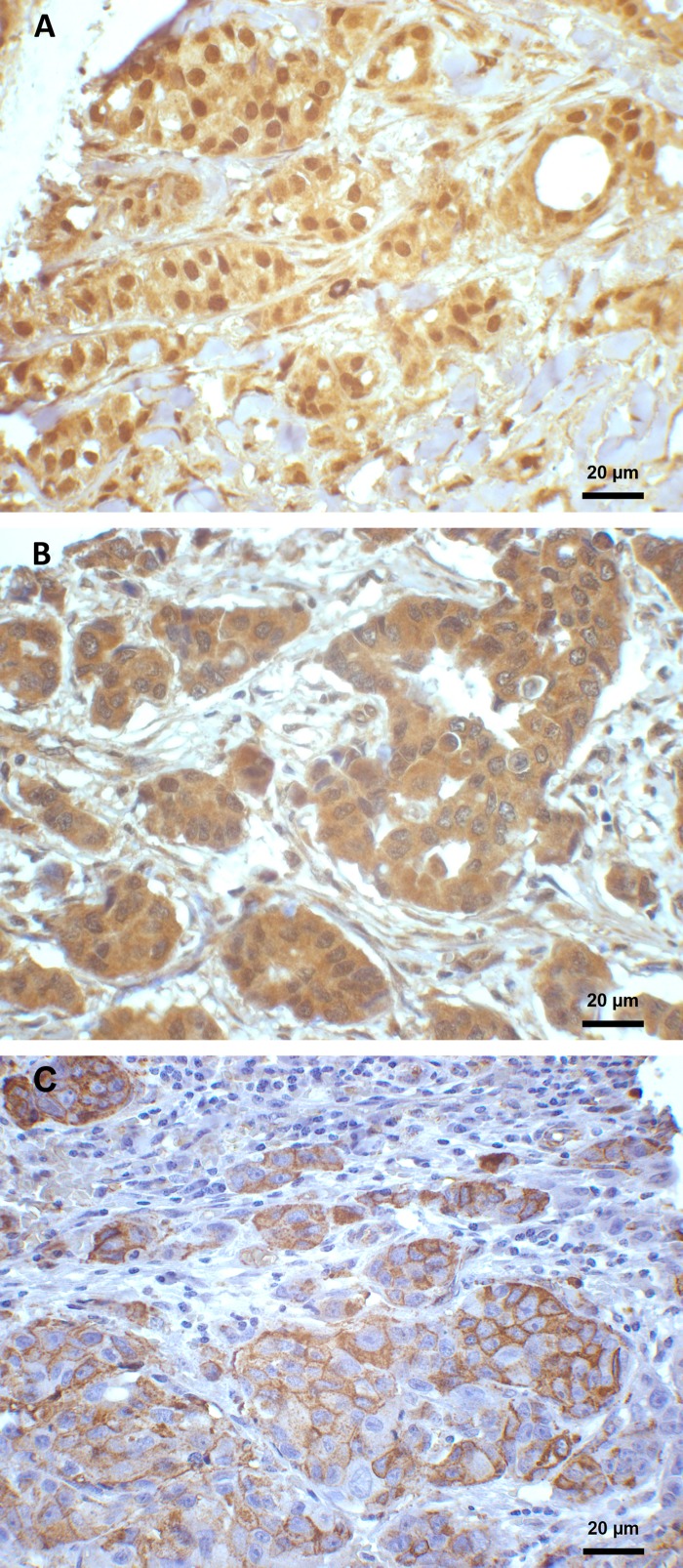
Immunohistochemical staining of HIF-1α, VEGF and Axl expression in breast cancer. (A) Moderate to strong HIF-1α positive staining located mainly in the nucleus (x400). (B) Moderate positive cytoplasmic expression of VEGF staining (x400). (C) Moderate to strong Axl positive staining, mainly membranous; weak cytoplasmic staining is also present (x400).

**Fig 2:**
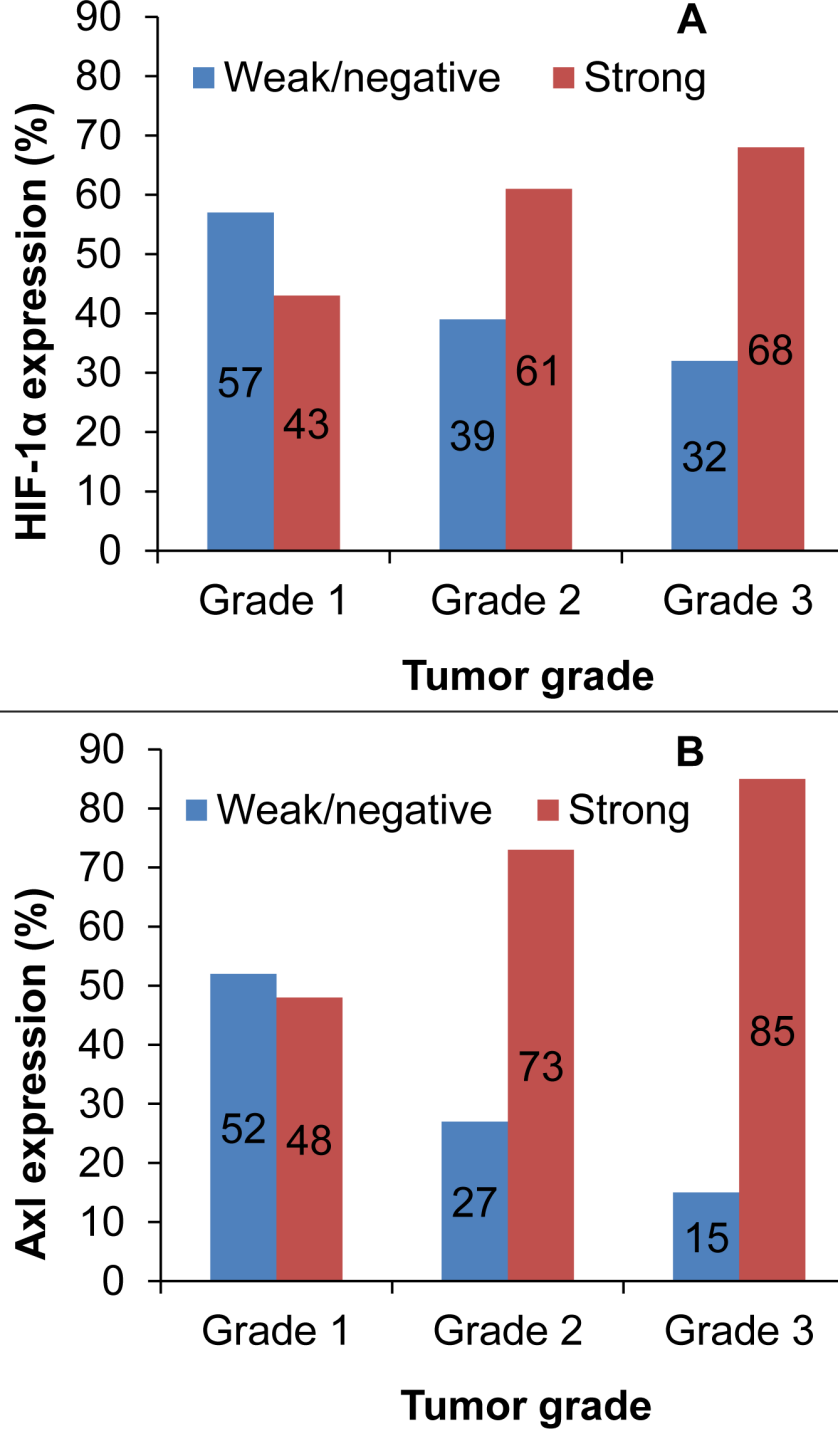
Distribution of HIF-1α expression (A) and Axl expression (B) by histologic grade. A majority of grade 2 and 3 tumors expressed HIF-1α (p = 0.037) and Axl (p<0.0005).

A majority (229/261; 88%) of the tumors were invasive carcinoma of no special type (NST) and of these, 134 (59%) were grade 3. In total, 165 of 261 tumors (63%) showed strong HIF-1α expression (**Fig 1**), and this was significantly associated with higher histologic grade (p = 0.037) (**Fig 2A**) and nuclear grade (p = 0.008). Furthermore, 188 of 243 tumors (77%) strongly expressed Axl, and this finding was associated with higher histologic grade as well (p<0.0005) (**Fig 2B**). Additionally, strong HIF-1α expression was significantly associated with strong Axl expression, high VEGF expression, high Ki-67 proliferative rate in tumor cells, and stronger p53 expression (**Table 2**).

**Table 2:** HIF-1α and associations with clinico-pathologic tumor and molecular characteristics.

Variable	HIF-1α Weak (SI = 0–2) (n; %) n = 96	HIF-1α Strong (SI = 3–9) (n; %) n = 165	OR 95% CI	P-value
**Age in years**				
<50	57 (41)	81 (59)	1	
≥50	33 (33)	67 (67)	1.4 (0.8–2.4)	NS
**Histologic type**				
Ductal carcinoma (NST)	80 (35)	149 (65)	1	
Others	16 (50)	16 (50)	0.5 (0.3–1.1)	NS
**Histologic grade**				
Grade 1	16 (57)	12 (43)	1	
Grade 2	32 (39)	51 (61)	2.1 (0.9–5.1)	0.086
Grade 3	48 (32)	102 (68)	2.8 (1.2–6.5)	0.011
**Nuclear grade**				
Grade 1	24 (57)	18 (43)	1	
Grade 2	38 (35)	72 (65)	2.5 (1.2–5.2)	0.011
Grade 3	34 (31)	75 (69)	3.0 (1.4–6.1)	0.003
**Mitotic count**				
0–6	22 (46)	26 (54)	1	
7–13	8 (18)	37 (82)	3.9 (1.5–10.1)	0.004
>13	66 (39)	102 (61)	1.3 (0.7–2.5)	NS
**Ki-67 proliferative rate**				
Low (<20.0%)	52 (45)	63 (55)	1	
High (≥20.0%)	41 (29)	101 (71)	2.0 (1.2–3.4)	0.007
**p53 expression**				
Low, SI = 0–4	74 (41)	105 (59)	1	
High, SI = 6–9	21 (27)	58 (73)	1.9 (1.1–3.5)	0.023
**ER expression**				
Positive (≥10%)	33 (34)	65 (66)	1	
Negative (<10%)	61 (38)	99 (62)	0.8 (0.5–1.4)	NS
**PR expression**				
Positive (≥10%)	19 (28)	49 (72)	1	
Negative (<10%)	73 (39)	115 (61)	0.6 (0.3–1.1)	NS
**HER2 expression**				
Negative, score 0–2+	74 (36)	133 (64)	1	
Positive, score 3+	20 (40)	30 (60)	0.8 (0.4–1.6)	NS
**Axl expression**				
Weak, SI = 0–2	29 (53)	26 (47)	1	
Strong, SI = 3–9	62 (33)	126 (67)	2.3 (1.2–4.2)	0.008

SI, staining index; NST, no special type; ER, estrogen receptor; PR, progesterone receptor; HER2, human epidermal growth factor receptor 2.

There was no significant association between HIF-1α or VEGF expression and basal markers, basal-like phenotypes, triple negative phenotype (ER−, PR−, HER2−) (**Table 3**), ALDH1 and c-KIT expression (**Table 4**). Strong Axl expression was associated with the basal-like subtype (odds ratio 3.0, 95% confidence interval 1.2–7.5, p = 0.016) compared to the luminal A subtype, whereas it showed only a weak association with P-cadherin (p = 0.045) and no association with the other basal markers (**S2 Table**).

**Table 3:** HIF-1α and VEGF associations with basal markers, basal-like phenotypes and subtypes.

Variable	HIF-1α Weak (SI = 0–2) (n; %) n = 96	HIF-1α Strong (SI = 3–9) (n; %) n = 165	P-value	VEGF Weak (SI = 0–2) (n; %) n = 55	VEGF Strong (SI = 3–9) (n; %) n = 130	P-value
**CK 5/6 expression**			NS			NS
Negative, SI = 0	79 (36)	138 (64)		45 (29)	110 (71)	
Positive, SI = 1–9	15 (38)	25 (62)		8 (29)	20 (71)	
**P-cad expression**			NS			NS
Negative, SI = 0–3	71 (38)	116 (62)		41 (31)	93 (69)	
Positive, SI = 4–9	25 (35)	47 (65)		13 (26)	37 (74)	
**EGFR expression**			NS			NS
Negative (≤1%)	70 (34)	134 (66)		44 (30)	102 (70)	
Positive (>1%)	24 (44)	30 (56)		10 (28)	26 (72)	
**BLP1**			NS			NS
Absent	80 (36)	141 (64)		45 (29)	111 (71)	
Present	13 (37)	22 (63)		8 (30)	19 (70)	
**BLP2**			NS			NS
Absent	77 (38)	128 (62)		45 (32)	98 (68)	
Present	18 (33)	37 (67)		9 (22)	32 (78)	
**BLP3**			NS			NS
Absent	76 (35)	140 (65)		47 (31)	105 (69)	
Present	18 (42)	25 (58)		7 (23)	24 (77)	
**BLP4 (CBP)**			NS			NS
Absent	71 (35)	132 (65)		42 (30)	100 (70)	
Present	22 (40)	33 (60)		12 (29)	29 (71)	
**BLP5**			NS			NS
Absent	68 (36)	121 (64)		42 (31)	92 (69)	
Present	25 (37)	43 (63)		12 (25)	37 (75)	
**TNP**			NS			NS
No	55 (36)	100 (65)		33 (31)	74 (69)	
Yes	38 (37)	65 (63)		21 (27)	56 (73)	
**Subtype**						
Luminal A	21 (38)	35 (62)		18 (38)	29 (62)	
Luminal B	16 (27)	43 (73)	NS	7 (19)	30 (81)	0.054
HER2	16 (44)	20 (56)	NS	8 (36)	14 (64)	NS
Basal-like (CBP)	22 (40)	33 (60)	NS	12 (29)	29 (71)	NS
Unclassified	16 (33)	32 (67)	NS	9 (25)	27 (75)	NS

CK, cytokeratin; P-cad, P-cadherin; TNP, triple negative phenotype; CBP, Core basal phenotype

**Table 4:** HIF-1α and VEGF association with Axl, ALDH1, and c-KIT markers of tumor angiogenesis.

Variable	HIF-1α Weak (SI = 0–2) n = 54	HIF-1α Strong (SI = 3–9) n = 128	P-value	VEGF Weak (SI = 0–2) n = 55	VEGF Strong (SI = 3–9) n = 130	P-value
**Axl expression**^a^			0.002^b^			0.001
Weak, SI = 0–2	20 (49)	21 (51)		20 (49)	21 (51)	
Strong, SI = 3–9	30 (24)	96 (76)		27 (21)	100 (79)	
**VEGF expression**						
Weak, SI = 0–2	29 (55)	24 (45)	<0.0005^b^			
Strong, SI = 3–9	24 (19)	103 (81)				
**ALDH1 expression**			NS			NS
Negative, SI = 0–2	31 (33)	62 (67)		32 (34)	63 (66)	
Positive, SI = 3–9	21 (24)	65 (76)		21 (24)	67 (76)	
**c-KIT expression**			NS			NS
Negative (<10%)	52 (30)	121 (70)		53 (30)	122 (70)	
Positive (≥10%)	1 (13)	7 (87)		0 (0)	8 (100)	
**MVD/mm**^**2**^						
Median	42.9	56.9	0.019^c^	44.0	57.0	0.007^c^
**pMVD/mm**^**2**^						
Median	0.4	0.9	0.027^c^	0.2	0.9	0.004^c^
**VPI (%)**						
Median	0.9	1.6	NS^c^	0.6	1.5	0.019^c^

SI, staining index; VEGF, vascular endothelial growth factor; MVD, microvessel density; pMVD, proliferating microvessel density; VPI, vascular proliferation index.

^a^shows association within the smaller cohort

^b^P value was calculated using Pearson’s χ^2^ test.

^c^P value was calculated using Mann-Whitney U test.

In addition, **Table 4** shows the median values for weak and strong expression categories of HIF-1α and VEGF. Tumors with strong HIF-1α expression had significantly higher MVD (p = 0.019) and pMVD (p = 0.027) than tumors with weak HIF-1α expression. Similarly, tumors with strong VEGF expression had significantly higher MVD (p = 0.007), pMVD (p = 0.004) and VPI (p = 0.019) than tumors with weak VEGF expression.

## Discussion

Previous studies have reported that HIF-1 is involved in breast tumorigenesis [15] by influencing growth rate and metastatic potential [8, 9] and consequently leading to an association with poor prognosis [17, 19, 40]. Our present findings indicate that this is also the case in African breast cancer since we found strong associations of HIF-1α expression with features of aggressive tumors. In particular, the association of HIF-1α with high histologic and nuclear grade extends previous literature indicating that the level of HIF-1α expression increases with the degree of malignancy [9, 15, 18].

The process of epithelial–mesenchymal transition (EMT), an important tumor progression program, can be activated by hypoxia, probably via a number of mechanisms such as HIF-1α signaling, in several human tumors and cell lines [6, 16, 23, 24]. At the same time, hypoxia is a well-known angiogenesis activator by production of HIF-1 transcriptional factors [6]. Hypoxia-inducible factors induce EMT by up-regulation of transcription regulators such as Twist, Snail, Slug and Zeb in several cell types [6, 24] during the EMT process. Also, the receptor tyrosine kinase Axl is known to be activated by epithelial–mesenchymal transition [25]. Interestingly, a study of prostate cancer has revealed that Axl expression was sustained in hypoxic tumor microenvironments [26]. Here, we report a strong co-expression of HIF-1α and Axl in this breast cancer cohort, supporting previous findings that Axl expression is increased within hypoxic tumor areas [26]. Notably, hypoxia has been found to prevent GAS6-mediated down-regulation of Axl in prostate cancer cells [26].

We here found that HIF-1α was strongly associated with VEGF expression in this series of African breast cancer, as also reported in other populations [15, 20, 40], although some did not find an association between HIF-1α and VEGF [18, 21]. Therefore, our results provide more support that HIF-1 is involved in angiogenesis by influencing VEGF transcription [10]. As a novel finding, HIF-1α expression was significantly associated with microvessel proliferation, a marker of activated angiogenesis [41]. In addition, strong VEGF expression was significantly associated with higher vascular proliferation, as well as overall vascular density, and our results support the importance of the VEGF pathway for angiogenesis in African breast cancer. Taken together, the findings indicate that anti-angiogenesis treatment could be an option in this population of aggressive and often late-stage breast cancer [3, 5]. Also, simultaneous targeting of VEGF and HIF-1 pathways would be a possibility supported by our data. Indeed, results from recent Phase I clinical trials with combined down-regulation of HIF-1α activity and a monoclonal antibody to VEGF are promising [42, 43]. Combinations with anti-Axl treatment [44] is another possibility indicated by our findings.

Additionally, a strong association between HIF-1α expression and Ki-67 proliferative rate in tumor cells was seen in our study. Our results, as well as previous studies [15, 18, 20, 40], support that HIF-1 factors promote major processes like tumor cell proliferation [6, 9] and support a hypothesis that Ki-67 proliferative rate might have a predictive role in classification of patients with VEGF positive high grade tumors for possible benefit from HIF-1α targeted therapy [45].

We found no significant associations between HIF-1α and ALDH1 or c-KIT expression. In experimental studies, HIF-1α was up-regulated in CSC-like cells that showed elevated ALDH1 expression [46], and ALDH1 was found to be associated with HIF-1α in locally advanced breast cancer [47]. Whereas studies in breast cancer cells have indicated that HIF-1α crucially regulates expression of stem cell factor (SCF), a c-KIT ligand [48], results on small-cell lung cancer cells indicated that activated SCF-c-KIT stimulated HIF-1α-mediated VEGF expression via PI3K/Akt activation [49].

Angiogenesis is related to both p53 and HER2 signaling pathways. Wild-type p53 suppresses VEGF transcription [50] and at the same time inhibits HIF-1α activity through targeting it for degradation. The decreased ubiquitylation of HIF-1α that follows loss of p53 function results in increased HIF-1α activity [51], which consequently promotes angiogenesis. It has been suggested that knowledge of both p53 mutation status and HIF-1α expression may influence choice of chemotherapy and HIF-1 inhibitors in cancer treatment [52]. In support of previous studies [20, 53], we found a significant association between p53 expression and strong HIF-1α staining. Further, experimental studies indicate that HER2 signaling is implicated in up-regulation of VEGF via mediators such as HIF-1α transcription factor to promote tumor angiogenesis [54, 55], and HIF-1α expression correlated with the HER2 subtype and HER2 positivity in breast cancers [18, 40, 56]. However, such associations were not found in our present study, in line with others [7, 19].

An inverse relationship between HIF-1α expression and ER status has been indicated in both human tumors and in experimental models [18, 57], although this relationship is still discussed. Our results showed no association between HIF-1α and ER expression in agreement with previous studies [17, 19]. More studies are required to determine the precise relationship between HIF-1α angiogenic drive and negative ER status.

Further, HIF-1α/VEGF is an important signaling pathway in breast cancer angiogenesis [58], while VEGF has been suggested as a candidate biomarker for the basal-like breast cancer [59], but we found no association between HIF-1α/VEGF and various basal markers in this cohort, in contrast to some other studies on basal-like tumors [60–62], triple negative breast cancers [63] and breast cancer cell lines [64]. HIF-1α expression was reported to correlate with P-cadherin expression in human breast carcinomas [61], while Gatza et al. [64] used six breast cancer cell lines to show that basal-like and triple negative cells expressed higher levels of HIF-1α mRNA compared to luminal cell lines. For VEGF expression, some results indicate that VEGF expression correlated with CK 5/6, and the basal-like subtype was more likely than luminal A tumors to express VEGF [60]. A similar finding was reported by Ribeiro-Silva et al. [62] in a smaller study although they used only a single marker CK5 positivity to define the basal-like subtype. On the contrary, lack of association between HIF-1α/VEGF and the basal-like phenotype or triple negative subtype in our study agrees well with previous findings in human carcinomas [65–67]. Thus, apart from methodological differences, our findings could suggest that additional angiogenic HIF-1 independent factors [68] are important for the angiogenic drive in African breast cancer. Martin et al. [4], using genome-wide mRNA expression profiles, noted that pathways related to angiogenesis might function differently between patients with African ancestry and Caucasians. Generally, we agree with Lindner et al. [69] that race is an important factor to consider when planning optimal therapeutic strategies for patients.

In conclusion, there is a frequent expression of HIF-1α in this series of breast cancer from an African population, which is significantly associated with strong Axl co-expression in addition to associations with other factors of poor prognosis like VEGF expression and increased angiogenesis, high tumor cell proliferation by Ki-67 rate, p53 expression, as well as high histologic tumor grade. Thus, HIF-1α and Axl as potential therapeutic targets in African breast cancer might be considered.

## Supporting Information

S1 Table Immunoreactivity evaluation criteria for the biomarkers in the present study. (DOCX)

S2 Table Axl and association with clinico-pathologic tumor and molecular characteristics. (DOCX)
