# Supplementary material for: Strong Expression of Hypoxia-Inducible Factor-1α (HIF-1α) Is Associated with Axl Expression and Features of Aggressive Tumors in African Breast Cancer
Source: PLoS One. 2016 Jan 13;11(1):e0146823. doi: 10.1371/journal.pone.0146823 (PMC4711940; doi:10.1371/journal.pone.0146823)
Supplement: S1 Table — (DOCX) [file pone.0146823.s001.docx]

**S1 Table. Immunoreactivity evaluation criteria for the biomarkers in the present study.**

| **Biomarker** | **Staining pattern** | **Cut-point** |
| --- | --- | --- |
| HIF-1α | Nuclear | Median SI; Weak (SI = 0–2), Strong (SI = 3–9) |
| Axl [1] | Cell membrane and cytoplasm | Median SI; Weak (SI = 0–2), Strong (SI = 3–9) |
| VEGF [1] | Cytoplasmic | Median SI; Weak (SI = 0–2), Strong (SI = 3–9) |
| ALDH1 [2] | Cytoplasmic | Median SI; Negative = SI <3, Positive = SI ≥3 |
| P-cadherin [3, 4] | Cell membrane and cytoplasm | Median SI; Negative (SI = 0–3), Positive (SI = 4–9) |
| p53 [2] | Nuclear | Upper quartile SI; Negative (SI = 0–4), Positive (SI = 6–9) |
| Cytokeratin 5/6 [3, 4] | Cell membrane and cytoplasm | Negative (SI = 0), Positive (SI = 1–9) |
| EGFR [5-7] | Cell membrane | Negative (≤1%), Positive (>1%) |
| ER [3] | Nuclear | At least 10% positive |
| PR [3] | Nuclear | At least 10% positive |
| c-KIT [6, 8] | Membrane and/or cell cytoplasm | Negative (<10%) Positive (≥10%) |
| HER2 [9] | Complete circumferential membrane staining | Negative (0–2+) Positive (3+) |
| Ki-67 proliferative rate [2] | Nuclear | Median value; < 20.0% = low, ≥ 20.0% = high |

1. Ahmed L, Nalwoga H, Arnes JB, Wabinga H, Micklem DR, Akslen LA. Increased tumor cell expression of Axl is a marker of aggressive features in breast cancer among African women. APMIS. 2015;123(8):688-696. doi: 10.1111/apm.12403. PubMed PMID: 26011102

2. Nalwoga H, Arnes JB, Wabinga H, Akslen LA. Expression of aldehyde dehydrogenase 1 (ALDH1) is associated with basal-like markers and features of aggressive tumours in African breast cancer. Br J Cancer. 2010;102(2):369-375. Epub 2009/12/17. doi: 10.1038/sj.bjc.6605488. PubMed PMID: 20010944

3. Nalwoga H, Arnes JB, Wabinga H, Akslen LA. Frequency of the basal-like phenotype in African breast cancer. APMIS. 2007;115(12):1391-1399. doi: 10.1111/j.1600-0463.2007.00862.x. PubMed PMID: 18184410

4. Collett K, Stefansson IM, Eide J, Braaten A, Wang H, Eide GE, et al. A Basal epithelial phenotype is more frequent in interval breast cancers compared with screen detected tumors. Cancer Epidemiol Biomarkers Prev. 2005;14(5):1108-1112. PubMed PMID: 15894660

5. Arnes JB, Collett K, Akslen LA. Independent prognostic value of the basal-like phenotype of breast cancer and associations with EGFR and candidate stem cell marker BMI-1. Histopathology. 2008;52(3):370-380. Epub 2008/02/14. doi: 10.1111/j.1365-2559.2007.02957.x. PubMed PMID: 18269588

6. Nalwoga H, Arnes JB, Wabinga H, Akslen LA. Expression of EGFR and c-kit is associated with the basal-like phenotype in breast carcinomas of African women. APMIS. 2008;116(6):515-525. doi: 10.1111/j.1600-0463.2008.01024.x PubMed PMID: 18754326

7. Dako. Guidelines for Interpreting EGFR pharmDx™ 2006 September, 29 [cited 2015 November, 11]. Available from:

<http://www.dako.com/no/28219_guidelines_for_interpreting_egfr_phamdx.pdf>.

8. Ulivi P, Zoli W, Medri L, Amadori D, Saragoni L, Barbanti F, et al. c-kit and SCF expression in normal and tumor breast tissue. Breast Cancer Res Treat. 2004;83(1):33-42. PubMed PMID: 14997053

9. Dako. HercepTest™ Interpretation Manual - Breast 2006 October, 12 [cited 2015 November, 11]. Available from:

<http://www.dako.com/no/28630_herceptest_interpretation_manual-breast_ihc_row.pdf>.
