# Supplementary material for: Strong Expression of Hypoxia-Inducible Factor-1α (HIF-1α) Is Associated with Axl Expression and Features of Aggressive Tumors in African Breast Cancer
Source: PLoS One. 2016 Jan 13;11(1):e0146823. doi: 10.1371/journal.pone.0146823 (PMC4711940; doi:10.1371/journal.pone.0146823)
Supplement: S2 Table — (DOCX) [file pone.0146823.s002.docx]

**S2 Table. Axl and association with clinico-pathologic tumor and molecular characteristics.**

| **Variable** | **Axl Low**  **(SI=0-2)**  **(n; %) n=55** | **Axl High**  **(SI=3-9)**  **(n; %) n=188** | **OR 95% CI** | **P value** |
| --- | --- | --- | --- | --- |
| **Age in years** |  |  |  |  |
| <50 | 33 (26) | 93 (74) | 1 |  |
| ≥50 | 18 (19) | 78 (81) | 1.5 (0.8 – 3.0) | NS |
| **Histologic type** |  |  |  |  |
| Ductal carcinoma (NST) | 37 (18) | 175 (82) | 1 |  |
| Others | 18 (58) | 13 (42) | 1.5 (0.07 – 0.3) | < 0.0005 |
| **Histologic grade** |  |  |  |  |
| Grade 1 | 14 (52) | 13 (48) | 1 |  |
| Grade 2 | 20 (27) | 55 (73) | 3.0 (1.2 – 7.4) | 0.017 |
| Grade 3 | 21 (15) | 120 (85) | 6.2 (2.5 – 14.9) | < 0.0005 |
| **Nuclear grade** |  |  |  |  |
| Grade 1 | 16 (41) | 23 (59) | 1 |  |
| Grade 2 | 27 (26) | 77 (74) | 2.0 (0.9 – 4.3) | 0.080 |
| Grade 3 | 12 (12) | 88 (88) | 5.1 (2.1 – 12.3) | < 0.0005 |
| **Mitotic count** |  |  |  |  |
| 0-6 | 17 (38) | 28 (62) | 1 |  |
| 7-13 | 11 (30) | 26 (70) | 1.4 (0.6 – 3.6) | NS |
| >13 | 27(17) | 134 (83) | 3.0 (1.5 – 6.3) | 0.002 |
| **Ki-67 proliferative rate** |  |  |  |  |
| Low (<20.0%) | 30 (28) | 77 (72) | 1 |  |
| High (≥20.0%) | 22 (17) | 111 (83) | 2.0 (1.1 – 3.7) | 0.032 |
| **p53 expression** |  |  |  |  |
| Low, SI = 0–4 | 47 (28) | 120 (72) | 1 |  |
| High, SI = 6–9 | 8 (11) | 66 (89) | 3.2 (1.4 – 7.2) | 0.003 |
| **ER expression** |  |  |  |  |
| Positive (≥10%) | 29 (31) | 65 (69) | 1 |  |
| Negative (<10%) | 26 (18) | 122 (82) | 2.1 (1.1 – 3.8) | 0.016 |
| **PR expression** |  |  |  |  |
| Positive (≥10%) | 14 (22) | 51 (78) | 1 |  |
| Negative (<10%) | 40 (23) | 135 (77) | 0.9 (0.5 – 1.8) | NS |
| **HER2 expression** |  |  |  |  |
| Negative, score 0–2+ | 48 (25) | 145 (75) | 1 |  |
| Positive, score 3+ | 7 (15) | 40 (85) | 1.9 (0.8 – 4.5) | NS |
| **CK 5/6 expression** |  |  |  | NS |
| Negative, SI = 0 | 48 (24) | 155 (76) | 1 |  |
| Positive, SI = 1–9 | 7 (18) | 31 (82) | 1.4 (0.6 – 3.3) |  |
| **P-cad expression** |  |  |  | 0.045 |
| Negative, SI = 0–3 | 44 (26) | 123 (74) | 1 |  |
| Positive, SI = 4–9 | 11 (15) | 64 (85) | 2.1 (1.0 – 4.3) |  |
| **EGFR expression** |  |  |  | NS |
| Negative (≤1%) | 46 (24) | 144 (76) | 1 |  |
| Positive (>1%) | 9 (18) | 42 (82) | 1.5 (0.7 – 3.3) |  |
| **BLP1** |  |  |  | NS |
| Absent | 48 (23) | 158 (77) | 1 |  |
| Present | 7 (21) | 26 (79) | 1.1 (0.5 – 2.8) |  |
| **BLP2** |  |  |  | NS |
| Absent | 47(25) | 144 (75) | 1 |  |
| Present | 8 (16) | 43 (84) | 1.8 (0.8 – 4.0) |  |
| **BLP3** |  |  |  | NS |
| Absent | 49 (24) | 152 (76) | 1 |  |
| Present | 6 (15) | 34 (85) | 1.8 (0.7 – 4.6) |  |
| **BLP4 (CBP)** |  |  |  | NS |
| Absent | 46 (24) | 144 (76) | 1 |  |
| Present | 9 (18) | 42 (82) | 1,5 (0.7 – 3.3) |  |
| **BLP5** |  |  |  | NS |
| Absent | 43 (24) | 134 (76) | 1 |  |
| Present | 12 (19) | 52 (81) | 1.4 (0.7 – 2.8) |  |
| **TNP** |  |  |  |  |
| No | 35 (24) | 111 (76) | 1 |  |
| Yes | 20 (21) | 75 (79) | 1.2 (0.6 – 2.2) |  |
| **Subtype** |  |  |  |  |
| Luminal A | 20 (39) | 31 (61) | 1 |  |
| Luminal B | 11 (19) | 47 (81) | 2.8 (1.2 – 6.5) | 0.019 |
| HER2 | 3 (9) | 31 (91) | 6.7 (1.8 – 24.7) | 0.002 |
| Basal-like (CBP) | 9 (18) | 42 (82) | 3.0 (1.2 – 7.5) | 0.016 |
| Unclassified | 11 (25) | 33 (75) | 1.9 (0.8 – 4.7) | NS |

NST, no special type; BLP1, concurrent ER−, HER2− and CK5+; BLP2, concurrent ER−, HER2− and P-cadherin+; BLP3, concurrent ER−, HER2− and EGFR+; BLP4, concurrent ER−, HER2− and CK5+ and/or EGFR+; BLP5, concurrent ER−, HER2− and positive for one or more basal markers (CK5, P-cadherin and EGFR). Luminal A: ER+, PR+, HER2−, Ki-67 < 20%; Luminal B: Luminal B HER2 negative (ER+, PR+, HER2−, Ki-67 ≥ 20%) and Luminal B HER2 positive (ER+, PR+, HER2+, any Ki-67); HER2 subtype: ER−, PR−, HER2+; basal-like subtype: ER−, PR−, HER2−, CK5/6+ and/or EGFR+; Unclassified: ER−, PR−, HER2−, CK5/6−, EGFR−.
